# Dietary Supplementation with GBE and TP Alleviates Heat Stress-Induced Lung Oxidative Damage in Broilers

**DOI:** 10.3390/ani16081206

**Published:** 2026-04-15

**Authors:** Xingyue Wu, Shuang Wu, Yuelong Chen, Lifang Si, Rui Zheng, Huaiyong Zhang, Siqiang Liu, Yanqun Huang, Wen Chen, Xuemeng Si

**Affiliations:** 1 Department of Companion Animal Science, Henan Agricultural University, Zhengzhou 450046, China; wuxy016@163.com (X.W.); 17637165775@163.com (S.W.); 18236192793@163.com (Y.C.); ruizheng1212@126.com (R.Z.); huaiyongzhang@henau.edu.cn (H.Z.); liusiqiang@henau.edu.cn (S.L.); hyanqun@aliyun.com (Y.H.); 2 College of Animal Science and Technology, Henan University of Science and Technology, Luoyang 471000, China; slif2004@163.com

**Keywords:** heat stress, broiler, pulmonary injury, Ginkgo biloba extract, tea polyphenols

## Abstract

Heat stress is a major environmental stressor in poultry production that reduces growth performance and induces oxidative stress, inflammatory responses, and lung tissue injury. This study investigated the protective effects of dietary supplementation with Ginkgo biloba extract and tea polyphenols against heat stress-induced lung injury in broilers. A total of 320 one-day-old broilers were reared under standard management conditions until 21 days of age. Subsequently, 300 birds with similar body weight were selected and randomly allocated into six groups: a thermoneutral control group, a heat stress group, a heat stress group supplemented with 300 mg/kg tea polyphenols, and three heat stress groups receiving 300 mg/kg tea polyphenols combined with 100, 300, or 600 mg/kg Ginkgo biloba extract. The analysis of the results showed that heat stress significantly reduced the average daily gain and feed intake while increasing the feed conversion ratio. It also markedly increased serum lactate dehydrogenase activity and malondialdehyde levels, while decreasing antioxidant-related indicators, indicating the occurrence of oxidative stress and inflammatory responses. Histological examination revealed lung injury characterized by alveolar wall thickening, epithelial cell shedding, and disruption of the endothelial barrier, accompanied by upregulated expression of heat shock proteins and inflammation-related signaling genes. Supplementation with tea polyphenols alone partially alleviated these changes. Notably, the combined supplementation of Ginkgo biloba extract and tea polyphenols exerted more pronounced protective effects, significantly enhancing antioxidant capacity, attenuating inflammatory responses, and maintaining pulmonary barrier integrity. Among the tested levels, the groups receiving 300 mg/kg Ginkgo biloba extract in combination with tea polyphenols showed the most evident improvements. These findings suggest that the combined use of these plant-derived extracts effectively mitigates heat stress-induced lung injury in broilers.

## Simple Summary

High environmental temperatures are a major challenge for the poultry industry, as heat reduces feed intake, suppresses growth, and impairs animal health. This study aimed to investigate whether two natural plant-derived bioactive substances, ginkgo leaf extract and tea polyphenols, could help broiler chickens cope with heat stress. Under high-temperature conditions, broilers showed significant reductions in body weight and feed intake, along with increased oxidative stress, inflammatory responses, and organ damage. In particular, accelerated respiration under heat exposure led to lung injury. After dietary supplementation with ginkgo leaf extract and tea polyphenols, growth performance improved and the adverse effects of heat were alleviated. The combined supplementation reduced indicators of inflammation and oxidative damage and helped protect the structure and function of lung tissue. Compared with supplementation of tea polyphenols alone, the combined use of the two plant-derived substances produced more pronounced protective effects. In this study, the most consistent benefits were observed when the diet contained 300 milligrams per kilogram of ginkgo leaf extract together with 300 milligrams per kilogram of tea polyphenols. These results suggest that combining such natural plant extracts may serve as a practical nutritional strategy to improve poultry health and production performance under hot environmental conditions.

## 1. Introduction

With the increasing severity of climate change caused by global warming, heat stress has become the primary environmental stressor affecting livestock and poultry production [1]. Modern commercial broilers, characterized by rapid growth and high feed efficiency, are especially susceptible to heat stress [2]. Heat stress is associated with significant economic losses in the poultry industry due to reduced growth performance, impaired feed efficiency, and increased mortality. Moreover, the increasing frequency of extreme heat events driven by climate change further exacerbates these challenges. The optimal temperature range for most poultry species is 18 °C to 20 °C [3]. Once the environmental temperature rises above 32 °C, most poultry exhibit pronounced heat stress accompanied by physiological dysfunction and metabolic disorders. Under such conditions, broilers display a marked increase in respiratory rate, which aggravates pulmonary pressure, induces lung tissue damage, and subsequently impairs the normal function of key organs such as the brain and gastrointestinal tract [4]. Meanwhile, heat stress also compromises poultry performance by inducing oxidative stress [5]. Under normal circumstances, the oxidant and antioxidant systems of poultry maintain the dynamic balance. However, excessive production of reactive oxygen species (ROS) or impairment of antioxidant defense system disrupts this balance and leads to oxidative stress [6,7]. Given the detrimental impact of heat stress on broiler production, developing effective mitigation strategies has become increasingly urgent. In recent years, nutritional regulation has emerged as a simple and efficient approach widely applied to alleviate the adverse effects of heat stress in poultry.

To mitigate the adverse effects of heat stress, various management and nutritional strategies have been proposed, among which dietary supplementation with antioxidants has attracted considerable attention due to its feasibility and cost-effectiveness [8]. Phytogenic feed additives, in particular, have gained increasing use in poultry production owing to their antioxidant, anti-inflammatory, and immunomodulatory properties. Ginkgo biloba extract (GBE), derived from the dried leaves of Ginkgo biloba, is rich in flavonoid glycosides and terpene lactones, and exhibits strong free radical-scavenging and circulatory-enhancing activities [9,10,11]. Clinically, GBE has been widely used to alleviate respiratory and cardiovascular disorders and acute lung injury [12,13]. In animal husbandry, it has also been applied as a plant-derived feed additive to improve growth performance and stress tolerance in poultry [14,15]. Tea polyphenols (TP) are polyphenolic compounds extracted from tea leaves, with catechins being the principal bioactive constituents [16]. TP exhibit strong antioxidant capacity and have been shown to alleviate heat stress-induced damage in broilers, partly by modulating heat shock protein (HSP) expression [17]. By scavenging free radicals and inhibiting the generation of H_2_O_2_ and ROS, TP effectively reduce oxidative stress-mediated cellular injury and contribute to improved poultry health [18,19]. Both GBE and TP have individually been shown to enhance heat tolerance, reduce inflammatory responses, and protect against tissue injury in poultry and other animals [20]. However, despite their complementary biological properties, limited information is available regarding the combined effects on heat stress-induced lung injury in broilers. Whether co-supplementation exerts synergistic or additive protective effects compared with single supplementation remains largely unexplored.

Heat stress-induced lung injury is closely associated with excessive inflammatory responses and disruption of the pulmonary air–blood barrier. The Toll-like receptors/nuclear factor kappa B (TLRs/NF-κB) signaling pathway play a central role in initiating innate immune responses and regulating inflammation by activation of downstream molecules such as myeloid differentiation primary response 88 (MYD88), interleukin-1 receptor-associated kinase 1 (IRAK1), and tumor necrosis factor receptor-associated factor 6 (TRAF6) [21,22,23]. Overactivation of this pathway elevates pro-inflammatory cytokines expression and exacerbates lung injury, whereas suppression of IRAK1 and TRAF6 can inhibit NF-κB activation and attenuate inflammatory damage [24]. Altered air–blood barrier permeability is a hallmark of lung injury [25]. The pulmonary air–blood barrier, composed of alveolar epithelial cells, the basement membrane, and capillary endothelial cells, restricts solute and fluid passage through tight junction-mediated physical barriers [26,27]. Disruption of this barrier increases pulmonary permeability, impairs gas exchange, and ultimately leads to respiratory dysfunction. Given the antioxidant and anti-inflammatory properties of GBE and TP, it is plausible that their combined supplementation may alleviate heat stress-induced lung injury by suppressing TLRs/NF-κB-mediated inflammation and preserving barrier integrity. Therefore, the present study aims to investigate the protective effects of dietary supplementation with graded doses of GBE in combination with a fixed level of TP on lung injury in heat-stressed broilers and to elucidate the underlying mechanisms, thereby providing a theoretical foundation for the application of combined phytogenic additives in poultry production. It was hypothesized that dietary supplementation with GBE and TP would alleviate heat stress-induced lung injury by attenuating inflammatory responses and preserving pulmonary barrier integrity, and that increasing levels of GBE under a constant TP background would exert dose-dependent protective effects.

## 2. Materials and Methods

### 2.1. Reagents

Ginkgo biloba extract (GBE) and tea polyphenols (TP) were used as feed additives in this experiment. GBE was purchased from Osaka Pharmaceutical Co., Ltd., (Osaka, Japan) and standardized to contain 24% ginkgo flavonoids and 6% terpene lactones. Tea polyphenols (TP) were obtained from Zhejiang Yinuo Biotechnology Co., Ltd. (Quzhou, Zhejiang, China) with a purity of 98% (batch number: 20220820). All reagents were stored in a dry and dark environment according to the manufacturers’ instructions prior to use.

### 2.2. Animals and Experimental Design

All experiments were carried out following the guidelines set by the Institutional Animal Care and Use Committees of Henan Agricultural University (Approval No. HENAU-2022-015) and Henan University of Science and Technology (Approval No. DK20230643), and were conducted in accordance with the Guide for the Care and Use of Laboratory Animals.

A total of 320 one-day-old male broiler chicks were obtained from a commercial hatchery. From day 1 to 21, birds were reared in a climate-controlled chamber at 25 ± 1 °C with 60% relative humidity and vaccinated according to the standard immunization program. At 21 days of age, 300 broilers with similar body weight were selected and randomly allocated into six treatment groups, with five replicates per group and ten birds per replicate: a thermoneutral control group (Control), a heat stress group (HS), a heat stress group supplemented with 300 mg/kg tea polyphenols (TP), and three heat stress groups receiving 300 mg/kg tea polyphenols combined with 100 (GBE100), 300 (GBE300), or 600 (GBE600) mg/kg Ginkgo biloba extract. For the Control group, ambient temperature was maintained at 25 °C, whereas broilers in the heat stress groups were exposed 35 °C for 12 h daily (08:00–20:00). The Control and HS groups received a basal diet, the composition and nutrient levels of which are presented in Table A1, while the treatment groups were supplemented with the designated additives. All broilers had ad libitum access to feed and water. Feed intake and body weight were recorded to calculate average daily feed intake (ADFI), average daily gain (ADG), and feed-to-gain (F/G) ratio.

### 2.3. Sample Collection

Sampling was performed at 35 and 42 days of age. One broiler was randomly selected from each replicate and euthanized for sample collection. Blood samples were collected, and serum was separated by centrifugation at 4000× *g* for 10 min at 4 °C. Lung tissue blocks (1 mm × 1 mm × 5 mm) were excised and fixed in glutaraldehyde for ultrastructural analysis. Additional lung samples were fixed in 4% paraformaldehyde for histological examination, while remaining portions were snap-frozen in liquid nitrogen and stored at −80 °C for subsequent analyses.

### 2.4. Morphological Analysis

Lung tissues fixed in paraformaldehyde were dehydrated, cleared, embedded in paraffin, and sectioned at a thickness of 4–6 μm. Sections were stained with hematoxylin and eosin (H&E) and examined under a light microscope (Olympus Corp., Tokyo, Japan) to assess lung morphological characteristics.

For transmission electron microscopy (TEM), lung tissues fixed in glutaraldehyde were post-fixed, dehydrated, embedded, and polymerized. Ultrathin sections (60 nm) were cut using an Ultracut Leica UC7 ultramicrotome (Leica Microsystems, Wetzlar, Germany), mounted on 150-mesh carbon-coated copper grids and stained with uranyl acetate and lead citrate. Ultrastructural features were observed using a transmission electron microscope.

Pulmonary injury was evaluated primarily by assessing alveolar structural integrity, inflammatory cell infiltration, hemorrhage, and epithelial cell morphology and arrangement. These parameters were integrated to determine the overall severity of lung damage.

### 2.5. Blood Biochemical Indexes

Serum concentration of tumor necrosis factor-α (TNF-α), interleukin-6 (IL-6), and corticosterone (CORT) were measured using ELISA kits (Boster Biological Technology Co., Ltd., Wuhan, China) according to the manufacturer’s protocol. Serum malondialdehyde (MDA) levels, activities of superoxide dismutase (SOD) and lactate dehydrogenase (LDH), and total antioxidant capacity (T-AOC) were measured using commercial assay kits (Nanjing Jiancheng Bioengineering Institute, Nanjing, China) following the manufacturer’s instructions.

### 2.6. Quantitative Real-Time PCR

Total RNA was extracted from lung tissues using TRIzol reagent (Nanjing Vazyme Biotech Co., Ltd., Nanjing, China). RNA concentration and purity were assessed with a micro-spectrophotometer (Thermo Scientific, Wilmington, DE, USA). First-strand cDNA was synthesized using a reverse transcription kit (Nanjing Vazyme Biotech Co., Ltd., China). Quantitative real-time PCR (RT-qPCR) was performed using SYBR Green Master Mix (Nanjing Vazyme Biotech Co., Ltd., China) on a real-time PCR detection system (Thermo Fisher Scientific, Waltham, MA, USA). Each reaction was conducted in a total volume of 20 μL containing cDNA template, SYBR Green mix, and gene-specific primers (Table 1). The thermal cycling conditions were: 95 °C for 30 s, followed by 40 cycles of 95 °C for 10 s and 60 °C for 30 s. Melting curve analysis was performed to confirm amplification specificity. Relative gene expression was calculated using the 2^−ΔΔCt method, with β-actin as the internal reference gene.

### 2.7. Statistical Analysis

All data were analyzed using IBM SPSS Statistics 26 software. Prior to analysis, data were tested for normality using the Shapiro–Wilk test and for homogeneity of variances using Levene’s test. When the assumptions were satisfied, differences among groups were analyzed by one-way ANOVA followed by Tukey’s post hoc test. If these assumptions were not met, appropriate data transformation or non-parametric tests were considered. Data were presented as mean ± SD, and statistical significance was set at *p* < 0.05.

## 3. Results

### 3.1. Effects of Dietary GBE and TP on Growth Performance in Heat-Stressed Broilers

Compared with the Control group, broilers exposed to heat stress exhibited a significantly increased F/G ratio (*p* < 0.05), accompanied by a significant reduction in ADG and ADFI (*p* < 0.05) (Table 2). However, dietary supplementation with GBE and TP markedly alleviated these heat stress-induced impairments in growth performance (*p* < 0.01). Among the treatment groups, the most pronounced improvements were observed in the GBE300 and GBE600 groups (Table 2).

### 3.2. Effects of Dietary GBE and TP on Serum Inflammation and Oxidative Status in Heat-Stressed Broilers

Heat stress significantly elevated serum TNF-α, IL-6, and CORT concentrations in the HS group on both days 35 and 42 (*p* < 0.01) (Table 3). These increases were significantly attenuated by dietary supplementation with GBE and TP (*p* < 0.05). Moreover, broilers receiving the combined GBE and TP treatments exhibited lower serum levels of those inflammatory markers compared with the TP group, with the most pronounced protective effects in the GBE300 and GBE600 groups.

Serum LDH activity and MDA levels were significantly increased in heat-stressed broilers on days 35 and 42 (*p* < 0.01), and these alterations were markedly alleviated by dietary supplementation (*p* < 0.05) (Table 3). Specifically, LDH activity in the GBE300 and GBE600 groups was significantly lower than that in the HS group (*p* < 0.01), whereas no significant differences in MDA levels were observed among the treatment groups. Compared with the Control group, the HS group exhibited a marked reduction in serum SOD activity and T-AOC (*p* < 0.01). Supplementation effectively mitigated these impairments, with the GBE300 and GBE600 groups showing greater improvements than the TP and GBE100 groups.

### 3.3. Effects of Dietary GBE and TP on Lung Histopathology in Heat-Stressed Broilers

**Figure 1:**
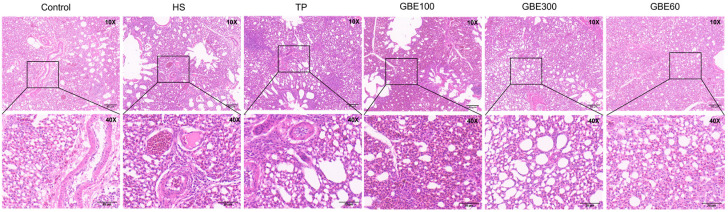
HE staining of lung tissue of each group at 35 days. Note: Upper panels: 10× objective magnification; lower panels: 40× objective magnification of the boxed regions. The degree of pathological injury in lung tissues was evaluated based on the integrity of alveolar structure, inflammatory cell infiltration in the pulmonary interstitium, extent of pulmonary congestion and edema, and integrity of the bronchial epithelium.

**Figure 2:**
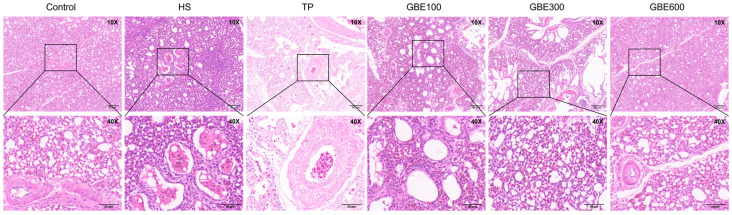
HE staining of lung tissue of each group at 42 days. Note: Upper panels: 10× objective magnification; lower panels: 40× objective magnification of the boxed regions. The degree of pathological injury in lung tissues was evaluated based on the integrity of alveolar structure, inflammatory cell infiltration in the pulmonary interstitium, extent of pulmonary congestion and edema, and integrity of the bronchial epithelium.

Histopathological examination revealed varying degrees of pulmonary congestion and hemorrhage in heat-stressed broilers on days 35 and 42 (Figure 1 and Figure 2), accompanied by widened alveolar septa, epithelial cell shedding, and structural damage to pulmonary microvascular endothelial cells. Hematoxylin and eosin (H&E) staining showed that dietary supplementation with GBE and TP markedly alleviated heat stress-induced lung injury at both time points. However, in the TP group, pronounced intrapulmonary hemolysis and infiltration of inflammatory cells within the alveoli were still evident. In contrast, the combined GBE and TP groups exhibited superior protective effects compared with TP alone. Notably, in the GBE300 and GBE600 groups, alveolar wall structures remained intact, and inflammatory cell infiltration was markedly reduced compared with the GBE100 group.

### 3.4. Effects of Dietary GBE and TP on Heat Shock Proteins and MLCK/MLC Expression in Heat-Stressed Broilers

**Figure 3:**
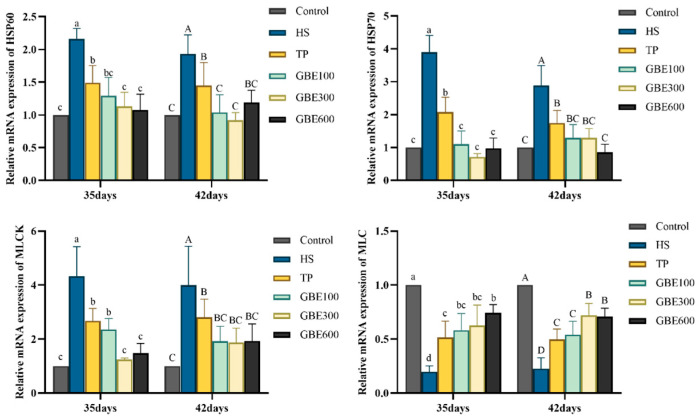
HSP60, HSP70, MLCK, MLC mRNA expression in lung tissue. Note: Different lowercase letters (a, b, c, d) indicate significant differences at 35 days (*p* < 0.05); different uppercase letters (A, B, C, D) indicate significant differences at 42 days (*p* < 0.05).

RT-qPCR analysis revealed that heat stress markedly upregulated HSP60 and HSP70 mRNA expression in lung tissues (*p* < 0.01) (Figure 3). This alteration was alleviated in all treatment groups, with greater reductions observed in the combined GBE and TP groups than in the TP-alone group, particularly at medium and high GBE doses.

Heat stress also significantly increased MLCK expression in lung tissues (*p* < 0.01), accompanied by a marked decrease in myosin light chain (MLC) expression (*p* < 0.01) (Figure 3). Compared with the HS group, MLCK expression was significantly reduced in all treatment groups (*p* < 0.05). At 35 days, the GBE300 group showed the most pronounced effect (*p* < 0.01), while at 42 days, all three combined-treatment groups displayed strong efficacy. For MLC expression, all treatment groups exhibited significantly higher levels compared with the HS group (*p* < 0.05), with the combined GBE and TP groups performing better than the TP group. Among them, the GBE600 group showed the most prominent effect at 35 days, whereas the GBE300 group was most effective at 42 days.

### 3.5. Effects of Dietary GBE and TP on TLRs/NF-κB Signaling in HEAT-Stressed Broilers

**Figure 4:**
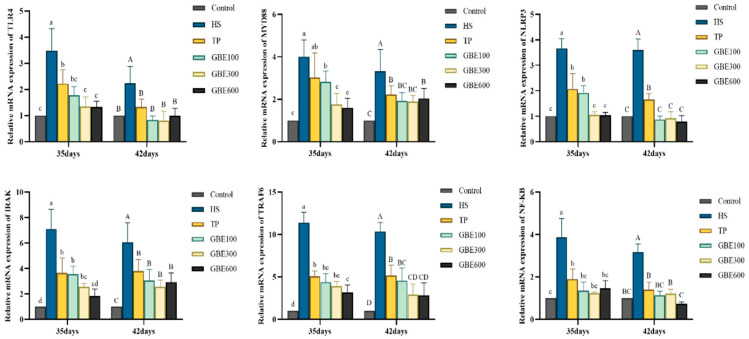
Expression of TLRs/NF-κB pathway-related factors in lung tissue. Note: Different lowercase letters (a, b, c, d) indicate significant differences at 35 days (*p* < 0.05); different uppercase letters (A, B, C, D) indicate significant differences at 42 days (*p* < 0.05).

The mRNA expression levels of TLRs/NF-κB-related factors, including TLR4, MYD88, NLRP3, IRAK, TRAF6, and NF-κB, were significantly upregulated in the HS group (*p* < 0.01) (Figure 4). These heat stress-induced increases were markedly attenuated by dietary supplementation, with the combined GBE and TP treatments showing greater reductions than the TP group at both time points. At 35 days, the GBE300 and GBE600 groups within the combined-treatment regimen exhibited notably lower mRNA expression levels of TLR4, MYD88, NOD-like receptor pyrin domain-containing protein 3(NLRP3), and IRAK compared with the GBE100 group. However, by 42 days, no significant differences were observed among the three combined-treatment groups (*p* > 0.01). At 35 days, the regulatory effects of the GBE100 group were comparable to those of the TP group for certain pathway factors. Nevertheless, with prolonged heat stress exposure, the combined-treatment groups demonstrated significantly lower expression levels of TLRs/NF-κB-related factors than the single-agent groups, with medium and high GBE doses showing the strongest effects.

### 3.6. Effects of Dietary GBE and TP on Pulmonary Air–Blood Barrier Ultrastructure in Heat-Stressed Broilers

**Figure 5:**
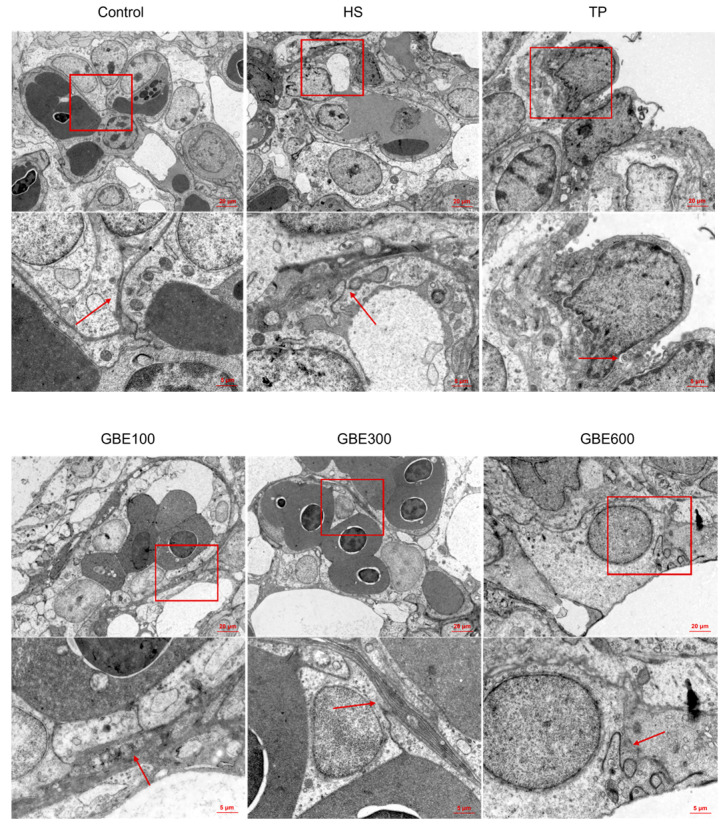
Transmission electron micrographs of lung tissue in groups at 35 days. The first row of images was acquired at a magnification of 2.0k, and the red box corresponds to the field of view shown in the second row (8.0k). The red arrow indicates the tight junctions between epithelial cells.

Transmission electron microscopy (TEM) analysis at 35 days of age (Figure 5) revealed that heat stress-induced pulmonary ultrastructural damage, including the disruption of tight junction structures between epithelial cells, enlargement of intercellular spaces, and loss of basement membrane integrity. These alterations were attenuated by dietary supplementation. However, abnormal epithelial cell morphology and widened intercellular gaps remained evident in the TP group. In contrast, broilers receiving combined GBE and TP supplementation exhibited relatively intact basement membranes and well-preserved tight junction structures.

**Figure 6:**
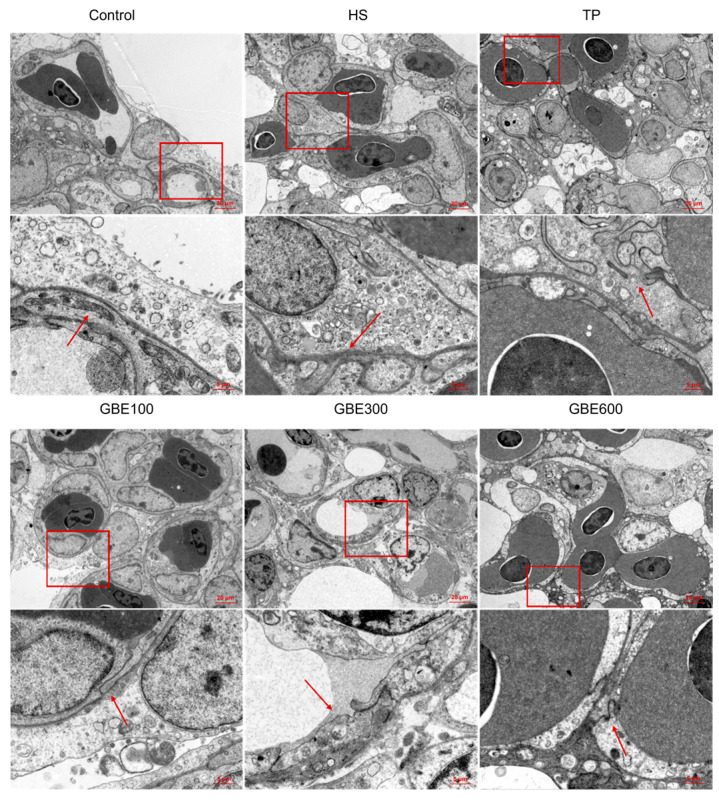
Transmission electron micrographs of lung tissue in groups at 42 days. The first row of images was acquired at a magnification of 2.0k, and the red box corresponds to the field of view shown in the second row (8.0k). The red arrow indicates the tight junctions between epithelial cells.

At 42 days (Figure 6), heat stress resulted in epithelial cell damage, disruption of tight junctions, and compromised basement membrane integrity. In the TP group, intercellular spaces were partially reduced, whereas tight junction structures remained impaired. These ultrastructural abnormalities were markedly improved in the combined treatment groups, with the GBE300 and GBE600 groups showing the most evident preservation of pulmonary air–blood barrier integrity.

## 4. Discussion

Heat exposure has a global impact on the livestock and poultry industry, exerting significant effects on animal health and production performance. Under high-temperature conditions, poultry and livestock reduce feed intake to lower metabolic heat production, which consequently results in reduced growth rates and production efficiency [28,29]. This represents a typical behavioral response of broilers to heat stress. Previous studies have demonstrated a pronounced negative correlation between ambient temperature and feed intake in broilers [30]. Under heat stress, nutrient allocation in animals undergoes substantial alterations: ingested nutrients are preferentially directed toward maintaining thermal homeostasis rather than supporting growth, thereby limiting nutrient utilization and ultimately reducing production performance and feed conversion efficiency in poultry [31]. In the present study, heat stress led to decreases in body weight, average daily gain, and average daily feed intake, while the feed-to-gain ratio increased in broilers. Importantly, dietary supplementation with GBE and TP improved the growth performance of heat-stressed broilers by alleviating inflammatory responses and mitigating oxidative stress, indicating that nutritional intervention effectively alleviated heat stress-induced growth impairment. These findings are consistent with previous observations that antioxidant-rich phytogenic additives contribute to improved performance under thermal stress conditions [32,33].

GBE has attracted increasing attention as a functional feed additive for alleviating heat stress in poultry. Bioactive constituents of GBE, such as flavonoids and phenolic compounds, have been reported to reduce body temperature, attenuate inflammatory responses, and enhance thermotolerance in animals [34,35]. These effects are thought to be closely associated with the antioxidant properties of flavonoids [36]. Similarly, tea polyphenols (TP), as plant-derived secondary metabolites, are widely recognized for their strong antioxidant properties and their ability to mitigate heat stress-induced damage in poultry [17,34,37]. Although both GBE and TP exhibit antioxidant effects, their molecular structures, mechanisms of action, and biological targets differ considerably. Ginkgolides, for example, act as platelet-activating factor (PAF) receptor antagonists, thereby indirectly reducing oxidative damage associated with platelet aggregation. They also protect mitochondrial function and preserve the activity of SOD2 [38]. Whereas TP primarily suppress lipid peroxidation and enhance SOD1 transcription via activation of the Nrf2 pathway [39]. Because these compounds target different reactive oxygen species and cellular compartments, their combined use may exert synergistic antioxidant effects, providing broader protection against heat stress-induced oxidative injury. This complementary mode of action may underlie the superior efficacy observed with combined supplementation compared with either additive alone.

Heat stress-induced tissue injury is widely recognized to result from excessive oxidative stress and inflammatory responses [40]. In the present study, heat stress markedly increased serum levels of pro-inflammatory cytokines TNF-α and IL-6, as well as CORT, indicating systemic inflammatory activation in broilers. MDA levels, which indirectly reflect the extent of lipid peroxidation and cellular damage, were elevated following heat stress [41,42]. Clinically, decreased LDH levels suggest reduced cellular and tissue damage, whereas increased SOD activity and T-AOC demonstrate enhanced antioxidant defenses, contributing to protection against heat stress-induced oxidative injury. Heat stress increased serum LDH activity while suppressing antioxidant indices such as SOD activity and T-AOC, suggesting extensive oxidative injury. Notably, dietary supplementation with GBE combined with TP effectively attenuated heat stress-induced changes in both inflammatory and oxidative biomarkers, demonstrating superior protective efficacy compared with TP supplementation alone. These findings highlight a close link between the antioxidant capacity of the combined treatment and its ability to suppress heat stress-induced inflammatory responses. When intracellular oxidative stress surpasses a certain threshold, it compromises the integrity and function of pulmonary cell membranes, thereby impairing respiratory function and destabilizing the immune system. Our previous study has demonstrated that heat stress can induce pulmonary congestion, hemorrhage and inflammatory infiltration [43]. Consistent with these findings, the present study revealed marked histopathological lung damage under heat stress, characterized by epithelial cell shedding, intercellular space enlargement, and intrapulmonary hemolysis. Notably, supplementation with GBE and TP significantly alleviated heat stress-induced pulmonary injury, with the combined treatment groups, particularly GBE300 and GBE600, showing more pronounced protective effects.

Under heat stress conditions, HSPs are rapidly induced and activate protective mechanisms; thus, their upregulation may contribute to the recovery and stabilization of damaged cells, thereby enhancing thermotolerance. HSPs, particularly HSP60 and HSP70, play critical roles in cellular protection and protein homeostasis under stress conditions [44]. However, excessive HSP activation may also amplify inflammatory signaling. Previous studies have shown that HSP70 can interact with TLR4, activating downstreamMYD88, IRAK and TRAF6, ultimately triggering NF-κB-mediated inflammatory cascades [45,46]. The findings of the present study are consistent with previous reports. Therefore, it is suggested that heat stress may induce lung injury in broilers by upregulating the expression of HSP60 and HSP70 in lung tissue, thereby activating the TLR4/NF-κB signaling pathway. Regarding the protective role of TP, earlier studies reported that they mitigate heat stress-induced inflammatory responses by suppressing inflammatory mediators and modulating inflammatory pathways, conferring protection against acute lung injury in mice [34]. Importantly, combined supplementation with GBE and TP more effectively suppressed these stress- and inflammation-related signaling pathways than TP alone, suggesting that attenuation of the HSP–TLR4/NF-κB axis contributes to the observed protection against lung injury. Taken together, the results suggest that the enhanced efficacy of GBE and TP may be associated with their joint suppression of stress-responsive signaling pathways.

Alterations in the permeability of the alveolar–capillary barrier represent the primary pathological change in lung injury [47,48]. MLCK, a calcium-dependent protein kinase, phosphorylates MLC, thereby modulating the interaction between actin and myosin. This process alters cytoskeletal tension and disrupts the dynamic balance among tight junctions, adherens junctions, and cytoskeletal contraction, ultimately increasing pulmonary microvascular endothelial cell permeability and impairing the integrity of the alveolar–capillary barrier [49,50]. In the present study, heat stress markedly upregulated MLCK expression while suppressing MLC expression in lung tissues, indicating impaired barrier integrity. Ultrastructural observations by transmission electron microscopy further confirmed that heat stress caused severe disruption of tight junctions, widened intercellular spaces, and basement membrane damage. Dietary intervention effectively reversed these alterations, with combined GBE and TP supplementation exerting greater regulatory effects than TP alone, particularly in the GBE300 and GBE600 groups. Collectively, these results suggest that the combined supplementation of GBE and TP may protect pulmonary barrier integrity under heat stress, with the MLCK–MLC pathway potentially involved, along with the stabilization of intercellular junctions. Although the present study yielded meaningful findings, the range of GBE supplementation levels was relatively narrow, and the underlying mechanisms require further validation. Future studies should explore a wider range of supplementation regimens and conduct more in-depth mechanistic analyses to better elucidate the long-term effects of this combined additive on growth performance and meat quality under commercial production conditions.

## 5. Conclusions

This study demonstrates that dietary supplementation with GBE and TP alleviates heat stress-induced inflammation, oxidative damage, and pulmonary injury in broilers. The combined use of GBE and TP exerted more pronounced protective effects than either additive alone, partly through inhibiting TLR4/NF-κB signaling and preserving air–blood barrier integrity. In addition to reducing systemic stress responses, the combination also contributed to improved pulmonary histology and stabilized intercellular junction structure. Among the tested dosages, 300 mg/kg GBE together with 300 mg/kg TP produced the most consistent benefits. These findings support the use of this combination as an effective nutritional strategy to mitigate heat stress in broiler production.

## Figures and Tables

**Table 1:** Gene primer sequences.

Gene	Forward Primer	Reverse Primer
β-actin	CCGCTCTATGAAGGCTACGC	CTCTCGGCTGTGGTGGTGAA
HSP60	GATGTGAAGTTCGGTGCGGA	ATGGTGACAGCTACGGCATC
HSP70	TGTGGCCTTCACCGATACAG	TGGGGTCATCATACTTGCGG
TLR4	CCAAACACCACCCTGGACTT	CCATGGAAGGCTGCTAGACC
TRAF6	TTCCCTGACGGTAAAGTGCC	ACAAGAAACCTGCCTCCTGG
IRAK	GGAGGTGCTCTGTGGAACTA	CACTGGCTGGTTGGGACTTC
NLRP3	TAGAGTACGCGGGTGAAGGA	CTGTGAAACTGCCCAACACG
MYD88	GAGGGATGATCCGTATGGGC	ACACGTTCCTGGCAAGACAT
NF-κB	ACACCACTGGATATGGCAGC	TCTTGCTTGGATCAGGCGTT
MLCK	CTCTGTCGGACCCGCTAC	CATCCCCCATGATGTGGACC
MLC	CACATACGCGCAATGTGGAG	CTTGTTTGGGTCTGCCAAGC

Abbreviations: β-actin: Beta-actin; HSP 60: Heat shock protein 60; HSP 70: Heat shock protein 70; TLR4: Toll-like receptor 4; TRAF6: tumor necrosis factor receptor-associated factor 6; IRAK: interleukin-1 receptor-associated kinase; NLRP3: NOD-like receptor pyrin domain-containing protein 3; MYD88: myeloid differentiation primary response 88; NF-κB: nuclear factor kappa B; MLCK: myosin light chain kinase; MLC: myosin light chain.

**Table 2:** Effects of dietary supplementation with Ginkgo biloba extract and tea polyphenols on growth performance.

Items	Group						*p*-Value
	Control	HS	TP	GBE100	GBE300	GBE600	
21 D BW, g	929.80 ± 48.75	942.40 ± 37.26	936.00 ± 35.55	921.60 ± 42.10	924.80 ± 52.89	918.20 ± 44.43	0.96
42 D BW, g	2708.80 ± 78.56 ^a^	2237.60 ± 67.08 ^c^	2471.40 ± 67.34 ^b^	2448.60 ± 68.71 ^b^	2505.80 ± 73.37 ^b^	2526.00 ± 52.35 ^b^	<0.01
21–42 days							
BWG (g)	1779.0 ± 134.84 ^a^	1295.2 ± 52.95 ^c^	1535.4 ± 39.83 ^b^	1527 ± 28.10 ^b^	1581 ± 131.38 ^b^	1607.8 ± 54.08 ^b^	<0.01
ADG (g d^−1^)	84.71 ± 6.42 ^a^	61.68 ± 2.52 ^c^	73.11 ± 1.90 ^b^	72.71 ± 1.34 ^b^	75.29 ± 6.26 ^b^	76.56 ± 2.58 ^b^	<0.01
ADFI (g)	135.71 ^a^	117.74 ^c^	128.69 ^b^	126.90 ^b^	129.05 ^ab^	130.60 ^ab^	<0.01
F/G	1.60 ± 0.12 ^c^	1.91 ± 0.08 ^a^	1.76 ± 0.05 ^b^	1.75 ± 0.03 ^b^	1.71 ± 0.14 ^bc^	1.71 ± 0.06 ^bc^	<0.01

Data are expressed as mean ± SD (*n* = 5); BW = Body weight; BWG = Body weight gain; ADG = Average daily gain; ADFI = Average daily feed intake; F/G = Feed-to-gain ratio. Different superscripts (a, b, c) in the same row indicate significant differences (*p* < 0.05).

**Table 3:** Effects of dietary supplementation with Ginkgo biloba extract and tea polyphenols on serum parameters in broilers.

Items	Control	HS	TP	GBE100	GBE300	GBE600	*p*-Value
35 days							
IL-6 (pg/mL)	15.66 ± 2.23 ^d^	71.19 ± 4.44 ^a^	31.68 ± 2.36 ^b^	24.73 ± 4.01 ^bc^	21.76 ± 3.23 ^cd^	19.09 ± 8.23 ^cd^	<0.01
TNF-a (pg/mL)	75.25 ± 4.15 ^c^	110.39 ± 7.88 ^a^	102.15 ± 6.79 ^a^	90.12 ± 5.24 ^b^	79.37 ± 2.65 ^bc^	79.96 ± 4.22 ^bc^	<0.01
CORT (ng/mL)	41.86 ± 1.42 ^d^	67.21 ± 1.67 ^a^	58.32 ± 2.63 ^b^	57.65 ± 3.13 ^b^	49.48 ± 2.64 ^c^	56.83 ± 3.04 ^b^	<0.01
LDH (U/L)	3152.73 ± 92.72 ^d^	5258.46 ± 281.77 ^a^	4031.66 ± 208.74 ^b^	4283.18 ± 206.27 ^b^	3447.51 ± 339.50 ^cd^	3676.03 ± 170.98 ^c^	0.015
SOD (U/mL)	132.64 ± 2.66 ^b^	103.05 ± 2.03 ^d^	119.48 ± 2.49 ^c^	122.37 ± 2.52 ^c^	136.09 ± 2.66 ^ab^	140.73 ± 2.15 ^a^	<0.01
MDA (nmol/mL)	1.36 ± 0.28 ^b^	2.62 ± 0.57 ^a^	1.31 ± 0.33 ^b^	1.34 ± 0.30 ^b^	1.19 ± 0.51 ^b^	1.36 ± 0.32 ^b^	<0.01
T-AOC (mM)	0.84 ± 0.08 ^a^	0.44 ± 0.06 ^c^	0.57 ± 0.09 ^b^	0.62 ± 0.09 ^b^	0.74 ± 0.10 ^a^	0.76 ± 0.09 ^a^	<0.01
42 days							
IL-6 (pg/mL)	9.11 ± 0.80 ^d^	47.91 ± 8.33 ^a^	26.94 ± 5.93 ^b^	23.50 ± 3.13 ^b^	12.33 ± 3.39 ^cd^	19.27 ± 4.56 ^bc^	<0.01
TNF-a (pg/mL)	84.18 ± 4.30 ^bc^	107.35 ± 8.75 ^a^	94.78 ± 4.71 ^b^	80.37 ± 3.37 ^c^	79.65 ± 3.73 ^c^	78.03 ± 5.85 ^c^	<0.01
CORT (ng/mL)	51.44 ± 1.82 ^c^	67.36 ± 1.64 ^a^	57.86 ± 2.33 ^b^	56.86 ± 2.28 ^b^	49.02 ± 2.01 ^c^	48.99 ± 2.19 ^c^	<0.01
LDH (U/L)	2875.45 ± 164.08 ^c^	4838.94 ± 223.76 ^a^	3612.46 ± 85.15 ^b^	3654.15 ± 248.35 ^b^	3137.85 ± 285.75 ^c^	2895.59 ± 324.15 ^c^	<0.01
SOD (U/mL)	133.46 ± 2.53 ^b^	105.69 ± 2.10 ^d^	123.70 ± 2.41 ^c^	126.88 ± 2.74 ^c^	134.77 ± 2.19 ^ab^	139.03 ± 2.14 ^a^	<0.01
MDA (nmol/mL)	0.88 ± 0.17 ^b^	2.01 ± 0.47 ^a^	1.37 ± 0.32 ^b^	0.90 ± 0.43 ^b^	1.01 ± 0.21 ^b^	1.07 ± 0.21 ^b^	<0.01
T-AOC (mM)	0.79 ± 0.10 ^a^	0.51 ± 0.06 ^d^	0.63 ± 0.03 ^c^	0.65 ± 0.07 ^bc^	0.75 ± 0.05 ^ab^	0.74 ± 0.06 ^ab^	<0.01

Data are expressed as mean ± SD (*n* = 5); IL-6 = Interleukin-6; TNF-a = tumor necrosis factor alpha; CORT = Corticosterone; LDH = Lactate dehydrogenase; SOD = Superoxide dismutase; MDA = Malondialdehyde; T-AOC: Total Antioxidant Capacity. Different superscripts (a, b, c, d) in the same row indicate significant differences (*p* < 0.05).

## Data Availability

Data are available from the authors upon request.

## Abbreviations

The following abbreviations are used in this manuscript:

ADFI	Average daily feed intake
ADG	Average daily gain
F/G	Feed-to-gain
CORT	Corticosterone
FCR	Feed conversion ratio
GBE	Ginkgo biloba extract
HSP	Heat shock protein
IRAK	Interleukin-1 receptor-associated kinase
LDH	Lactate dehydrogenase
MDA	Malondialdehyde
MLCK	Myosin light chain kinase
MLC	Myosin light chain
NLRP3	NOD-like receptor pyrin domain-containing protein 3
ROS	Reactive oxygen species
MYD88	Myeloid differentiation primary response 88
SOD	Superoxide dismutase
T-AOC	Total antioxidant capacity
TLR4	Toll-like receptor 4
NF-ΚB	Nuclear factor kappa B
TP	Tea polyphenols
TRAF6	Tumor necrosis factor receptor-associated factor 6

## Appendix A

**Table A1:** Experimental Diet Composition and Nutrients (as-fed basis, %).

Ingredients (%)	1–21 d	21–42 d
Corn	55.00	59.37
Soybean meal	34.00	31.90
Soybean oil	4.50	5.00
Limestone	1.00	1.23
Dicalcium phosphate	1.70	1.50
L-lysine	0.20	0.11
DL-methionine	0.35	0.27
Salt	0.30	0.30
Vitamin premix ^1^	0.03	0.03
Mineral premix ^2^	0.20	0.20
70%Choline chloride	0.15	0.09
Calculated nutrients		
Metabolizable energy (kcal/kg)	3200	3100
Crude protein	22.00	19.00
Calcium	0.90	0.90
Total phosphorus	0.63	0.56
Available phosphorus	0.40	0.35
Lysine	1.20	1.00
Methionine	0.50	0.46
Methionine + cystine	0.90	0.80
Threonine	0.65	0.60
Tryptophan	0.22	0.20

Note: ^1^ Vitamin premix provided per kilogram of diet: Vitamin A, 12,000 IU; Vitamin D3, 2500 IU; Vitamin E, 20 IU; menadione, 1.3 mg; thiamin, 2.21 mg; riboflavin, 7.8 mg; nicotinamide, 40 mg; calcium pantothenate, 16.5 mg; pyridoxine HCl, 4 mg; biotin, 0.04 mg; folic acid, 1.2 mg; Vitamin B12, 0.015 mg. ^2^ Mineral premix provided per kilogram of diet: iron, 80 mg; copper, 8.0 mg; manganese, 110 mg, zinc 65 mg; iodine, 1.1 mg; selenium, 0.3 mg.
